# Supplementary material for: An online training platform for SPECT imaging technology utilizing three-dimensional modeling
Source: PLoS One. 2026 Feb 12;21(2):e0323153. doi: 10.1371/journal.pone.0323153 (PMC12900319; doi:10.1371/journal.pone.0323153)
Supplement: S2 Text — (PDF) [file pone.0323153.s002.pdf]

Annex 2

1．Other imaging domains included in the platform

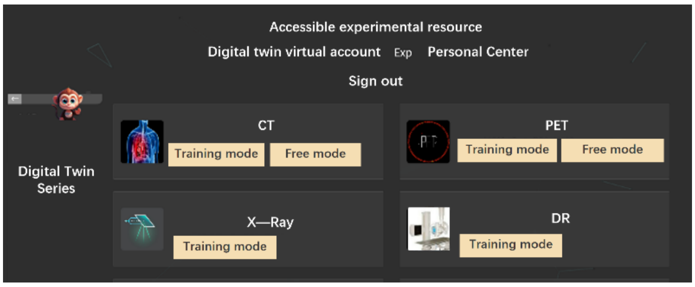

2. Other applied universities

Other applied universities 1：Nanchang Medical College

202202001143	Nanchang Medical College		SPECTSim	train	93	93	100	2024-10-28 14:4···	26.7min
202202001169	Nanchang Medical College		SPECTSim	train	93	93	100	2024-10-28 14:4···	27.5min
202202001167	Nanchang Medical College		SPECTSim	train	100	100	100	2024-10-28 14:4···	3.1min
202202001070	Nanchang Medical College	202202001070	SPECTSim	train	93	93	100	2024-10-28 14:4···	24.9min
202202001112	Nanchang Medical College		SPECTSim	train	83	83	100	2024-10-28 14:4···	25.3min
202202001135	Nanchang Medical College	202202001135	SPECTSim	train	78	78	100	2024-10-28 14:4···	21.7min
202202001168	Nanchang Medical College		SPECTSim	train	93	93	100	2024-10-28 14:4···	16.7min
202202001149	Nanchang Medical College	202202001149	SPECTSim	train	93	93	100	2024-10-28 14:4···	13.4min
202202001231	Nanchang Medical College		SPECTSim	train	93	93	100	2024-10-28 14:3···	24.6min
202202001227	Nanchang Medical College	202202001227	SPECTSim	train	100	100	100	2024-10-28 14:3···	3.5min
202202001167	Nanchang Medical College		SPECTSim	train	92	92	100	2024-10-28 14:3···	14.7min
202202001227	Nanchang Medical College	202202001227	SPECTSim	train	100	100	100	2024-10-28 14:3···	5.5min
202202001227	Nanchang Medical College	202202001227	SPECTSim	train	87	87	100	2024-10-28 14:2···	4.3min
202202001106	Nanchang Medical College	202202001106	SPECTSim	train	81	81	100	2024-10-28 14:2···	4.7min
202202001183	Nanchang Medical College	202202001183	SPECTSim	train	100	100	100	2024-10-28 14:2···	3.4min
202202001190	Nanchang Medical College		SPECTSim	train	100	100	100	2024-10-28 14:2···	4.3min
202202001227	Nanchang Medical College	202202001227	SPECTSim	train	92	92	100	2024-10-28 14:2···	2.9min
202202001183	Nanchang Medical College	202202001183	SPECTSim	train	100	100	100	2024-10-28 14:1···	4.7min
15879196212	Nanchang Medical College		SPECTSim	train	47	47	100	2024-10-26 10:1···	3.4min
202202001220	Nanchang Medical College		SPECTSim	train	81	81	100	2024-10-26 09:5···	3.1min
202202001207	Nanchang Medical College		SPECTSim	train	2	2	100	2024-10-26 09:4···	0.1min

Other applied universities 2：Ordos Institute of Technology

20228410031	Ordos Institute of Technology	SPECTSim	train	100	100	100	2024-11-26 11:0···	3.4min
20228410019	Ordos Institute of Technology	SPECTSim	train	100	100	100	2024-11-26 11:0···	2.7min
20228410049	Ordos Institute of Technology	SPECTSim	train	71	71	100	2024-11-26 11:0···	2.4min
20210010072	Ordos Institute of Technology	SPECTSim	train	63	63	100	2024-11-26 11:0···	2.5min
20228410017	Ordos Institute of Technology	SPECTSim	train	100	100	100	2024-11-26 11:0···	3min
20213110007	Ordos Institute of Technology	SPECTSim	train	100	100	100	2024-11-26 11:0···	2.7min
20228410036	Ordos Institute of Technology	SPECTSim	train	93	93	100	2024-11-26 11:0···	3.3min
20228410025	Ordos Institute of Technology	SPECTSim	train	100	100	100	2024-11-26 11:0···	3min
20228410041	Ordos Institute of Technology	SPECTSim	train	100	100	100	2024-11-26 11:0···	3.4min
20228410009	Ordos Institute of Technology	SPECTSim	train	100	100	100	2024-11-26 11:0···	2.3min
20228410035	Ordos Institute of Technology	SPECTSim	train	97	97	100	2024-11-26 11:0···	3.6min
20228410034	Ordos Institute of Technology	SPECTSim	train	88	88	100	2024-11-26 11:0···	2.7min
20228410042	Ordos Institute of Technology	SPECTSim	train	100	100	100	2024-11-26 11:0···	2min
20228410007	Ordos Institute of Technology	SPECTSim	train	100	100	100	2024-11-26 11:0···	3.6min
20228410016	Ordos Institute of Technology	SPECTSim	train	96	96	100	2024-11-26 10:5···	2.6min
20228410011	Ordos Institute of Technology	SPECTSim	train	100	100	100	2024-11-26 10:5···	2.6min
20218110015	Ordos Institute of Technology	SPECTSim	train	93	93	100	2024-11-26 10:5···	2.5min
20228410030	Ordos Institute of Technology	SPECTSim	train	100	100	100	2024-11-26 10:5···	2.9min
20228410023	Ordos Institute of Technology	SPECTSim	train	65	65	100	2024-11-26 10:5···	3.3min
20210010003	Ordos Institute of Technology	SPECTSim	train	97	97	100	2024-11-26 10:5···	4.8min
20210010081	Ordos Institute of Technology	SPECTSim	train	93	93	100	2024-11-26 10:5···	3.8min
20228410022	Ordos Institute of Technology	SPECTSim	train	100	100	100	2024-11-26 10:5···	2.4min

Other applied universities 3：Suzhou Vocational Health College

85220012	Suzhou Vocational Health College	SPECTSim	train	83	83	100	2025-01-03 21:3···	7.7min
23110517	Suzhou Vocational Health College	SPECTSim	train	100	100	100	2025-01-03 17:5···	3.7min
23110617	Suzhou Vocational Health College	SPECTSim	train	100	100	100	2025-01-03 17:5···	3.1min
23110617	Suzhou Vocational Health College	SPECTSim	train	78	78	100	2025-01-03 17:4···	3.0min
85220012	Suzhou Vocational Health College	SPECTSim	train	93	93	100	2025-01-03 17:4···	3.5min
85220012	Suzhou Vocational Health College	SPECTSim	train	100	100	100	2025-01-03 17:4···	3.1min
23110617	Suzhou Vocational Health College	SPECTSim	train	93	93	100	2025-01-03 17:3···	8.1min
85220012	Suzhou Vocational Health College	SPECTSim	train	90	90	100	2025-01-03 17:3···	3.8min
85220012	Suzhou Vocational Health College	SPECTSim	train	87	87	100	2025-01-03 17:3···	3.8min
85220012	Suzhou Vocational Health College	SPECTSim	train	100	100	100	2025-01-03 17:2···	3min
23110626	Suzhou Vocational Health College	SPECTSim	train	93	93	100	2025-01-03 17:2···	3.8min
85220012	Suzhou Vocational Health College	SPECTSim	train	93	93	100	2025-01-03 17:1···	3.6min
85220012	Suzhou Vocational Health College	SPECTSim	train	93	93	100	2025-01-03 17:1···	3.8min
85220012	Suzhou Vocational Health College	SPECTSim	train	90	90	100	2025-01-03 17:0···	3.7min
85220012	Suzhou Vocational Health College	SPECTSim	train	100	100	100	2025-01-03 16:5···	15.8min
85220012	Suzhou Vocational Health College	SPECTSim	train	90	90	100	2025-01-03 16:3···	7min
23110634	Suzhou Vocational Health College	SPECTSim	train	100	100	100	2025-01-03 16:3···	2.2min
23110634	Suzhou Vocational Health College	SPECTSim	train	91	91	100	2025-01-03 16:2···	1.4min
85220012	Suzhou Vocational Health College	SPECTSim	train	90	90	100	2025-01-03 16:2···	5.1min
23110624	Suzhou Vocational Health College	SPECTSim	train	97	97	100	2025-01-03 16:2···	2.4min
85220012	Suzhou Vocational Health College	SPECTSim	train	100	100	100	2025-01-03 16:2···	5min
23110605	Suzhou Vocational Health College	SPECTSim	train	100	100	100	2025-01-01 23:0···	2.3min
