# Supplementary material for: An online training platform for SPECT imaging technology utilizing three-dimensional modeling
Source: PLoS One. 2026 Feb 12;21(2):e0323153. doi: 10.1371/journal.pone.0323153 (PMC12900319; doi:10.1371/journal.pone.0323153)
Supplement: Attachment — Submitted filename: Response to Reviewers.docx [file pone.0323153.s004.docx]

**Response to Reviewers**

Dear Editor and Reviewers:

Thank you for your insightful feedback on this article. I will address the concerns of the article sequentially from the perspectives mentioned below.

**1 Editor's queries**

**Question 1. Please ensure that your manuscript meets PLOS ONE's style requirements, including those for file naming.**

Answer: The manuscript has been revised to conform to the style guidelines specified by "PLOS ONE". Please refer to the " Revised Manuscript with Track Changes" or " Manuscript -New " for further details.

**Question 2.** **Please ensure that you have an ORCID iD and that it is validated in Editorial Manager.**

Answer:The ORCID iD of the corresponding author has been duly added and verified in "Editorial Manager".

**Question 3. Please state what role the funders took in the study. If the funders had no role, please state: "The funders had no role in study design, data collection and analysis, decision to publish, or preparation of the manuscript."If this statement is not correct you must amend it as needed.Please include this amended Role of Funder statement in your cover letter; we will change the online submission form on your behalf.**

Answer:①The primary supporting project for this article is the "Research and Practice of Innovation and Entrepreneurship Education and Teaching Reform in Hebei Province's Universities in 2023 (2023CXCY068)". The project content aligns seamlessly with this article, focusing on the development and operation of a virtual experimental platform for nuclear medicine. This platform is designed to enhance the innovation and entrepreneurship training of professionals specializing in medical imaging technology. The findings of this article will be derived from the outcomes of the project.② The research concepts and program code for this study were supplied by the "Hebei Province Medical Science Research Project in 2022 (20220971)".③ This article's teaching innovation design and practice are founded on the project titled "Research and Practice of Higher Education Teaching Reform in Hebei Province (2022GJJG149)", a 2022 Education and Teaching Research Project from Hebei Medical University (2022YBZD-4, 2022YBPT-8). ④ Data analysis for this article is provided by the project "2023 College Students' Innovation Experiment Plan of Hebei Medical University (USIP2023337)".Consequently, all the aforementioned elements are pivotal to the content of this article and should be preserved.

**Question 4. Please amend either the title on the online submission form (via Edit Submission) or the title in the manuscript so that they are identical.**

Answer: Modified the title on the online submission form (by editing the submission) or in the manuscript, such that it is identical to the title in the revised manuscript.

**Question 5.** [**Please include captions for your Supporting Information files at the end of your manuscript, and update any in-text citations to match accordingly.**](http://track.editorialmanager.com/CL0/http:%2F%2Fjournals.plos.org%2Fplosone%2Fs%2Fsupporting-information./1/010f0196ee3d69f8-29bd965d-ffa2-4d26-b773-594911ec7270-000000/qp7-JFFpJTe5Yze5xqSl4-BnuO-1ow-a_RROfoM7leU=212)

Answer: The supporting information file's title and content are provided at the conclusion of the manuscript, as outlined on page 34 of the " Revised Manuscript with Track Changes" within the "Supporting Information" section.

**Question 6. Please review your reference list to ensure that it is complete and correct.。**

Answer：The references have been meticulously reviewed, and both the format and quantity of references have been appropriately revised in the "Revised Manuscript with Track Changes " on page 28, within the "References" section.

**2** **Questions Raised by Reviewer 1**

**Question 1.** **Please justify that this number of authors is really necessary? And where did they contribute?**

Answer :The online training platform for SPECT imaging technology, developed by our research team, represents a collaborative effort among universities, hospitals, and private enterprises. University instructors Shang Dandan, Qiao Lihua, Zhao Ruibin, and Qin Ruiping brought innovative concepts, pragmatic solutions, course execution, and authored articles to the project. Hospital nuclear medicine technologists Guo Xiaorui, Lei Xinkun, and Shang Hua supplied clinical data, constructed scenes, and designed cases. Wang Hongzhi, a professor at East China Normal University and chairman of Shanghai Peiyun Educational Technology Co., Ltd., alongside enterprise engineer Xia Tian, offered technical assistance, including system debugging and maintenance. Current students Fang Zikun, Shou Luqi, and Qin Yiwen contributed to the analysis and feedback on teaching practice data. In essence, this research encompassed the efforts of various stakeholders, including multiple institutions, educators, students, and company staff, spanning from development to instructional implementation. Consequently, every author has played a pivotal role. Please refer to the“Author Contributions”section, which has been added on page 28 of the“Revised Manuscript with Track Changes”.

**Question 2. Reduce old references and do not exceed 20.**

Answer：The number of outdated references has been suitably reduced to a maximum of 20, while the overall quantity of references has been augmented to 41, as indicated on page 28 "References" in the " Revised Manuscript with Track Changes ".

**Question 3. Do not write paragraphs that are too long or too short, try to maintain order.**

Answer：Paragraphs of lesser length were amalgamated and excessively long sentences were judiciously excised. This can be observed in the "Six Major Scenario Modules" section on pages 9-11, the "Process model" section from pages 11-12, and in the " Revised Manuscript with Track Changes " where longer paragraphs have been condensed on pages 11 and 14.

**Question 4. Your statement of the problem is a little weak. Explain in one paragraph why this research is important and necessary.**

Answer：The online training platform developed by our team for SPECT imaging technology addresses the high risks and costs associated with SPECT equipment, as well as the challenges of implementing experimental classes on campus. This platform offers a departure from traditional teaching constraints, allowing students to engage in educational practice without the limitations of time, space, or frequency, thereby accommodating their individualized needs. The platform's design is supported by training data which indicates that the overall sample score distribution aligns with a normal distribution. Furthermore, the positive slopes of individual sample scores suggest that the frequency of training sessions has a beneficial impact on students' personalized development. The integrated dynamic evaluation system provides constructive feedback on teaching outcomes, thereby achieving the objectives of nuclear medicine education in terms of personalized student cultivation, innovative thinking, and the development of an "early clinical" mindset.

**Question 5.** **Why did you choose Vietnam?**

Answer：It appears to be an operational error; the study was conducted in the People's Republic of China instead of Vietnam as originally intended. **Question 6.** **Your article is international. Can you achieve some generalization in the findings from a limited number of countries and individuals? Explain this a little bit about this issue as well.**

Answer：An online training platform for Single-Proton Emission Computed Tomography (SPECT) examination technology, incorporating AI assistant functions and featuring seven typical disease examinations, could not be located in international data sets. This absence is consistent with the computer software copyright associated with this study. The comprehensive teaching innovation model, from the conceptualization and development of the online training platform to its implementation in pedagogical practice, has not been previously observed in related SPECT teaching methodologies. Thus, this study possesses significant originality.

**Question 7.** **Completely and several times to strengthen the structure of the journal and observe all its issues.**

Answer：The paper has been restructured in accordance with your request and is now presented within the " Revised Manuscript with Track Changes ".

**Question 8.** **Explain whether the sample is sufficient for the research population and can it be strengthened?**

Answer：To date, over 550 students from our institution have engaged in teaching practice on this platform, fully addressing the research objectives. This practice is executed in a phased manner, with class-specific instruction. Owing to space constraints within the article, data analysis was confined to students from a particular class in a specific semester. Furthermore, three additional universities are utilizing this platform. For further details, please see Attachment 2.
**Question 9.** I**n my opinion, strengthen the qualitative section of the article because it will provide readers with the existing attractions.**

Answer：The "Abstract" on page 3 and the "Discussion" on page 23 of the " Revised Manuscript with Track Changes " have been revised by incorporating qualitative speech.
**Question 10.** **The limitations of the research are not included in the conclusion.**

Answer：The limitations have been excised from the conclusion section and relocated to the " Revised Manuscript with Track Changes " in the " Conclusion " section on page 25.
**Question 11.** **Add a section titled Discussion and compare all the research with previous research.**
Answer：A "Discussion" section has been incorporated on page 23 of the " Revised Manuscript with Track Changes ". Additionally, a comparative analysis with previous research has been included. The data relevant to this comparison is presented on page 17 and 23 of the " Revised Manuscript with Track Changes " within the Comparative Analysis of Pre-class and Post-class Test Scores.

**Question 12.** **There needs to be some direct promise in the article, which has made the text very sweet, but it is more literary than scientific.**
Answer：In the " Revised Manuscript with Track Changes ", we have incorporated future planning commitments and application expansion commitments into the “Conclusion” section on page 25.

**Question 13.** **The sixth research did not study new research in this field and this makes your article look like an old article.**

Answer：This study has secured a copyright for the computer software utilized in the research, as outlined in Appendix 1(S1 File), underscoring the novelty of the project. The development and implementation of an online training platform, specifically designed for educational purposes, represents the most comprehensive and systematic teaching system globally for conducting SPECT virtual experiments within an academic setting. Consequently, this study is both innovative and of significant practical value.
**Question 14. The article is too short, or you were in a hurry, or you could not spend the time as much as you would like.**

Answer：The supplementary content is comprehensively outlined in the " Revised Manuscript with Track Changes ": on page 2 within the "Abstract" section; page 15, where "Table 3" now includes data for "2024-2025(2)"; page 17 and 23, which features an added section titled "Comparative Analysis of Pre-class and Post-class Test Scores"; page 18, showcasing updated "Table 4" data; and page 22 and 25, where three new subsections have been introduced: "(4) The influence of customized training sessions on student evaluation scores", "Course Satisfaction Survey", and page 25" Conclusion".
**Question 15. Ten references are not enough for an original article, I think you should increase it to 40.**
Answer：The quantity of references has been augmented to a total of 42, as indicated within the "References" section on page 28 of the " Revised Manuscript with Track Changes ".

**Question 16. Please provide a timeline of the research process.**

Answer：June 2020 - December 2020: The university, enterprise, and hospital collaborated to sign an agreement, determining the research plan, including platform design, data collection, virtual experimental scenario modules, and operational process module design, etc.

January 2021 - February 2022: The initial development of the SPECT examination technology online training platform was completed, and it was piloted during the 2021-2022 (1) semester.

March 2022 - July 2022: The SPECT inspection technology online training platform was officially developed and completed, and was approved for computer software copyright, officially being put into educational practice.

August 2022 - July 2023: Iterative optimization of the SPECT examination technology online training platform, organization and analysis of teaching practice data.

August 2023 - December 2023: A provincial first-class undergraduate virtual simulation experimental teaching course based on this platform was applied for and approved.

Jan 2024–Mar 2025: Writing of papers related to the study, completion and submission of articles..

**3 Questions Raised by Reviewer 2**

**Question 1.** **It would have been good if the authors could elaborate why a control group was not used or comparative data against traditional teaching methods was done.**

Answer：In this study, a control group was not established, given the potential for individual students to attribute their performance outcomes either to the prohibition of using the training platform or its utilization. Instead, the research employed a comparative approach, examining the difference in knowledge mastery between pre-class and post-class assessments. Refer to page 17 and 23, "Comparative Analysis of Pre-class and Post-class Test Scores" in the " Revised Manuscript with Track Changes " for detailed insights, particularly Figure 9 data. In this context, the pre-class assessment serves as a proxy for traditional teaching methodologies, whereas the post-class evaluation represents innovative teaching approaches. Consequently, the study does incorporate control data.
**Question 2.** **While quantitative performance is analyzed, student perceptions, satisfaction, and engagement are only briefly mentioned. Please elaborate on these findings as well.**
Answer：The data from the satisfaction survey has been incorporated into the "Revised Manuscript with Track Changes " on page 23 and 25, within the section titled "Course Satisfaction Survey." This can be seen in Figure 13.
**Question 3.** **The manuscript would benefit from English editing to simplify the writing. Some sections (e.g., tables, explanations of AI functionality) are overly verbose or technically dense for a general readership.**
Answer：The section titled "Check point" in Table 2 (page 12) has been deleted, and the section labeled "Check name" in Table 3 (page 15) has been modified. Additionally, the feature 2 of "AI " on page 11 has been removed.All of them can be seen from the "Revised Manuscript with Track Changes ".
**Question 4.** **What extent the platform could be generalized to other imaging modalities or clinical areas?**

Answer：①The platform is currently integrated with imaging fields such as PET and CT (refer to Annex 2 for specifics). It is projected that within the subsequent five years, it will be extendable to the SPECT/CT imaging field, and within a decade, to the SPECT/MR and other related imaging fields.② Currently, the platform is not only being used by our own institution but has also been expanded to three other medical colleges (see Attachment 2 for details). Within the next five years, it is expected that the platform will cater to undergraduate or standard-training students from 10 medical colleges or affiliated hospitals. Within a decade, the target is to extend this service to 50 medical colleges or affiliated hospitals. This application promotion has been added to the "evised Manuscript with Track Changes " on page 25 in the "Conclusion" section.

These are my comprehensive responses. I eagerly await your feedback.

Best wishes!

Dandan Shang

2025.6.29
