# Supplementary material for: An online training platform for SPECT imaging technology utilizing three-dimensional modeling
Source: PLoS One. 2026 Feb 12;21(2):e0323153. doi: 10.1371/journal.pone.0323153 (PMC12900319; doi:10.1371/journal.pone.0323153)
Supplement: Attachment — Submitted filename: Response to Reviewers-2.docx [file pone.0323153.s005.docx]

**Response to Reviewers**

Dear Editor and Reviewers:

Thank you for your insightful feedback on this article. I will address the concerns of the article sequentially from the perspectives mentioned below.

**1 Editor's queries**

**Question 1.** Please include the following items when submitting your revised manuscript:A 'Response to Reviewers'.A 'Revised Manuscript with Track Changes'.A 'Manuscript'.

**Answer:** The initial draft has been thoroughly revised in accordance with the requested modifications. Please refer to the “Response to Reviewers-2“， " Revised Manuscript with Track Changes-2" and " Manuscript-2" for further details.

**Question 2.** If you would like to make changes to your financial disclosure, please include your updated statement in your cover letter.

**Answer:** I have included a statement of financial disclosure in “cover letter-2”.

**Question 3.**While revising your submission, please upload your figure files to the Preflight Analysis and Conversion Engine (PACE) digital diagnostic tool, PACE helps ensure that figures meet PLOS requirements.

**Answer:**I have upload my figures files to the Preflight Analysis and Conversion Engine (PACE) digital diagnostic tool. I have resubmitted the figure files.

**Question 4.**The PLOS Data policy requires authors to make all data underlying the findings described in their manuscript fully available without restriction.

**Answer:** All experimental data have been placed in file 2,“Practice data for all students” . Protocols.io has been alerted, and they are currently generating laboratory protocols. If the experimental designs and all associated data are must to be deposited with Protocols.io, these newly generated protocols will supersede file 2 in due course.

**2** **Questions Raised by Reviewer 3**

**Question 1.** The introduction is too short. The text in the “Platform construction” subsection would be better as part of the introduction. It does not explain the methods used by the authors?

**Answer:** The content from the subsection titled "Platform construction" has been incorporated into the introduction, and the references suggested by the review experts have been included. This can be found in lines 46-72 of the “Revised Manuscript with Track Changes-2”.

**Question 2.** I think that Figure 2 needs an improvement and probably a correction. Is the “User experience feedback” the evaluation part? I did not understand the sentence “The formative evaluation of the operator can be relayed to the background, thereby optimizing the system model further.” Is the operator the student? Is the formative evaluation the assessment of the knowledge acquired by the student? These are aspects that should be in the conceptual design framework.

**Answer:** Figure 2 has been updated，as depicted in Fig 2. "User experience feedback" refers to the evaluation section where operators, which could include students or teachers, participate in a virtual experiment. The data presented in this article is a statistical analysis derived from a population of students within a school setting who have had the opportunity to experience the experiment. The formative assessment focuses on evaluating the students' acquisition of knowledge and their ability to correctly and proficiently operate the experiment. It is also an evaluation of the platform functions, which helps the platform optimize iterations.

**Question 3.** Figure 4 is described in the text, referring to numbers for each module. However, the figure itself does not contain the numbers used to identify each module. Also, some expressions are confusing: what is “chairs of landmarks”? Could you use a more specific case for a body weight detector, or are there different such devices? It would be interesting to have images of all modules. What is a ”calling button”?.

**Answer:**. Figure 4 has been updated to incorporate numerical labels for each module, and the module identifiers within the figure have been harmonized with those used in the text. There are no “chairs of landmarks” in the article, only “waiting chairs” and “landmarks”. The weight detector is a standard scale (not a specialised medical one), as can be seen in the following Figure 6. It has been changed to " a weighing scale", as can be seen at line 159 of the “Revised Manuscript with Track Changes-2”. The “mumber calling button” is the button pressed by the doctor to call out the next patient number in the waiting room, as can be seen in Figure 6.The figure 6 has been incorporated at line 169 of the of the “Revised Manuscript with Track Changes-2”. Furthermore, the manuscript incorporates "Figure 8. The waiting room" to facilitate a more intuitive presentation of the content within this platform，as shown at line 174 of the “Revised Manuscript with Track Changes-2”.

Figure 6 The pre-examination preparation room

**Question 4.** As for Table 2, is “The injection room is filled of medicine” really a “Mode of Injection”?**.**

**Answer:** The translation provided is inaccurate. The correct interpretation should be "administering drugs to patients in an injection room," which refers to an "injection mode" of administration that is intravenous. Changes have been made to Table 2, line 184 of the “Revised Manuscript with Track Changes-2”.

**Question 5.** The description starting at line 222 and figure 8 is very confusing. There is no description of Figure 8. Only the list of the stages. Since this is an example, it should be complete and described as a usage scenario. Then, there is a description of the characteristics of the process model, which seems to be out of place. Probably it would be better to move this text to the conceptual design framework section because it is the design rationale of the platform.

**Answer:** The operating environment of the original Fig. 8 (now Fig. 10) is elaborated, and the contents in the operating process are elaborated and sorted out, as shown in lines 185 to 214 of the “Revised Manuscript with Track Changes-2”. In addition, this part of the content was added to the conceptual design framework section, as shown in Fig. 2. **Question 6.** If 550 students used the platform, why present only data from 2023-2024?

**Answer:** The SPECT platform undergoes annual optimizations and iterative updates, resulting in minor variations in statistical data collection each year. During its pilot phase, the system solely included training mode data, omitting assessment mode data. By the 2023-2024-1 semester, although data for both training and assessment modes was available, the platform only computed average scores for the training mode, excluding scores from the inaugural training session, thus rendering the data statistics incomplete. It was only in the 2023-2024-2 semester that the platform achieved optimal performance, leading to more comprehensive data statistics. Consequently, the original manuscript chose data from the most recent year as a benchmark. Nevertheless, following expert recommendations, statistical analyses were performed on data from the two latest semesters, specifically 2023-2024-2 and 2024-2025-2. These datasets, derived from the platform's latest iteration and boasting identical data formats, are apt for synchronous analysis. Refer to lines 285-306 and Figure 12 for further details，as in “Revised Manuscript with Track Changes-2”. It is important to note that, due to the lack of a mandatory requirement for the training mode, there were data omissions identified in the records of two students who may have bypassed the training phase and proceeded directly to the assessment phase. As a result, the total amount of data collected from the training mode is slightly less than that gathered from the assessment mode, accounting for the missing data from these two students.

**Question 7.** Are the differences between pre-test and post-test scores statistically significant? The authors report only means and standard deviation.

**Answer:** The difference between the pre-class and post-class tests is notable, with the average score of the latter increasing by 12.45%. Furthermore, the post-class test exhibits a larger median and a smaller variance compared to the pre-class test. Please refer to Table 4 for more details. This table has been newly incorporated. see lines 282 of the “Revised Manuscript with Track Changes-2”. Here, in addition, with the optimization of the teaching reform model, only the 2024-2025-2 semester has introduced the ternary classroom of intelligent course teaching. Therefore, the pre-class test and post-class test raw data only contain the data of 129 people in this semester. The reason for this data is also explained in the text, see lines 240 to 242 of the “Revised Manuscript with Track Changes-2”

**Question 8.** As far as I understood, the students followed the three-element classroom approach presented from lines 263-286 of the “Revised Manuscript with Track Changes-2”. The authors mention that the pre-test was applied before the tool was used. So, the students have already engaged in the “pre-class guidance”. On the other hand, the post-test was applied right after the use of the training tool. It is not clear how many times they could perform the simulation during the “interactive learning phase”, and if all students performed the same number of times the simulation tasks.

**Answer:** The simulation training is not constrained by a set number of attempts; therefore, during the interaction phase, students may undertake as many training sessions as they require based on their individual needs. This continues until the students feel they have thoroughly mastered the SPECT operation skills and knowledge points. As a result, the frequency of simulation training varies among students. This approach effectively caters to the personalized requirements of the students. This can be seen in lines 117-118 or lines 325 and 380.
**Question 9.** As a minor comment, please check lines 283-284. What are “Wanqianxing” and “Rain Classroom”?

**Answer:** “Wanqianxing” and “Rain Classroom” are two distinct teaching tools predominantly utilized for classroom assessments, data analytics, and real-time interactions. This section has been omitted in the latest version,as can be seen in lines 261-265 of the “Revised Manuscript with Track Changes-2”; kindly refer to the most recent draft for precise information.
 Furthermore, I have carefully reviewed and revised the unsuitable descriptions present in the text, as outlined in “Revised Manuscript with Track Changes-2”.

These are my comprehensive responses. I eagerly await your feedback.

Best wishes!

Dandan Shang

2025.10.2
