# Supplementary material for: An online training platform for SPECT imaging technology utilizing three-dimensional modeling
Source: PLoS One. 2026 Feb 12;21(2):e0323153. doi: 10.1371/journal.pone.0323153 (PMC12900319; doi:10.1371/journal.pone.0323153)
Supplement: Attachment — Submitted filename: Response to Reviewers-3.docx [file pone.0323153.s006.docx]

**Response to Reviewers**

Dear Editor and Reviewers:

Thank you for your insightful feedback on this article. I will address the concerns of the article sequentially from the perspectives mentioned below.

**1 Editor's queries**

**Question 1.** Please include the following items when submitting your revised manuscript:A 'Response to Reviewers'.A 'Revised Manuscript with Track Changes'.A 'Manuscript'.

**Answer:** The initial draft has been thoroughly revised in accordance with the requested modifications. Please refer to the “Response to Reviewers-3“， " Revised Manuscript with Track Changes-3" and " Manuscript-3" for further details.

**Question 2.** If you would like to make changes to your financial disclosure, please include your updated statement in your cover letter.

**Answer:** There added a fund (2024CHYB-48) in my financial disclosure. The statement of financial disclosure can be seen in “cover letter-3”.

**Question 3.** 1. If the reviewer comments include a recommendation to cite specific previously published works, please review and evaluate these publications to determine whether they are relevant and should be cited. There is no requirement to cite these works unless the editor has indicated otherwise.

**Answer:** The articles recommended by the reviewers were reviewed, confirming their relevance to this study and suitability for citation. See Ref. 2 under "References", as can be seen at lines 50 and 414.

**Question 4.** Please review your reference list to ensure that it is complete and correct. If you have cited papers that have been retracted, please include the rationale for doing so in the manuscript text, or remove these references and replace them with relevant current references. Any changes to the reference list should be mentioned in the rebuttal letter that accompanies your revised manuscript. If you need to cite a retracted article, indicate the article’s retracted status in the References list and also include a citation and full reference for the retraction notice.

**Answer:** The references no longer include citations to retracted or controversial articles . That is, references 3 and 4 were removed, as can be seen at lines 50 and 416–420. The citation numbers in existing references have been adjusted accordingly to maintain consistency with the revised numbering system.

**Question 5.** If applicable, we recommend that you deposit your laboratory protocols in protocols.io to enhance the reproducibility of your results.

**Answer:** The laboratory protocol has been deposited in protocols.io. Protocol Integer ID:228888.

Private link: https://www.protocols.io/private/7D8871759FCF11F0A1320A58A9FEAC02.

**Or** practice data for all students is provided in S2 File.

**2** **Questions Raised by Reviewer 3**

**Question 1.** The Introduction was improved. The authors kept the text in the “Platform construction” subsection, although it does not explain methods.

**Answer:** The main technical approaches for constructing the platform were incorporated into the “Introduction”. This can be found in lines 55 to 58 of the “Revised Manuscript with Track Changes-3”. Furthermore, the detailed technical methods are presented in lines 126 to 133 and table 1.

**Question 2.** I suggest eliminating the title “Principles of Construction and Technical Parameters” and keeping the text as part of “Platform Construction”. This small change will make the section “Platform Construction” really about the techniques used to build it.

**Answer:** The title "Principles of Construction and Technical Parameters" has been deleted and the text is keeping as part of “Platform Construction”.All changes have been completed. This can be found in lines 113 of the “Revised Manuscript with Track Changes-3”.

**Question 3.** An additional correction in the section “Platform construction” is to correct the references in the sentence “Over the past decade, several educators and practitioners, including Jonathan Cooper [9], Tao Shaoneng[10], Oliver A. Meyer [11], and Lawson AP [12], …..” These articles have multiple authors. The authors should just write “Over the past decades, several authors [9,10,11, 12] have ….”.

**Answer:** The changes of this sentence have been completed. This can be found in lines 94 to 96 of the “Revised Manuscript with Track Changes-3”.

**Question 4.** Figure 2 has been improved a lot. However, you can simplify the caption by replacing the current one with “Model Design Concept Diagram: Prerequisites, human-computer interaction, and feedback optimization. ”

**Answer:** The caption for Figure 2 has been simplified. This can be found in lines 118-120 and 539-541 of the “Revised Manuscript with Track Changes-3”.

**Question 5.** Figure 4 has been improved with the numbers, as has its description in the text. However, there still are “chairs of landmarks” in the figure. I think they are waiting chairs as the authors explained in the response letter. So, the figure needs to be corrected.

**Answer:** The “chairs of landmarks” has changed into “Waiting chairs” and “Landmarks”,as can be seen in figure 4.

**Question 6.** The authors made some adjustments in the section but did not add demographic information: for example, the reader does not know basic statistics about age, sex and previous experience with VR. These are common data that one usually adds when reporting experiments.

**Answer:** This paper provides comprehensive details regarding the study participants' demographic characteristics (e.g., age range, gender) and prior VR experience. This can be found in lines 219 to 223 of the “Revised Manuscript with Track Changes-3”.

Furthermore, I have carefully reviewed and revised the unsuitable descriptions present in the text, as outlined in “Revised Manuscript with Track Changes-3”.

These are my comprehensive responses. I eagerly await your feedback.

Best wishes!

Dandan Shang

2025.11.21
