# Supplementary material for: An online training platform for SPECT imaging technology utilizing three-dimensional modeling
Source: PLoS One. 2026 Feb 12;21(2):e0323153. doi: 10.1371/journal.pone.0323153 (PMC12900319; doi:10.1371/journal.pone.0323153)
Supplement: Attachment — Submitted filename: Response to Reviewers-4.docx [file pone.0323153.s007.docx]

**Response to Reviewers**

Dear Editor and Reviewers:

Thank you for your insightful feedback on this article. I will address the concerns of the article sequentially from the perspectives mentioned below.

**1 Editor's queries**

**Question 1.** Please include the following items when submitting your revised manuscript:a 'Response to Reviewers'.a 'Revised Manuscript with Track Changes'.a 'Manuscript'.

**Answer:** The manuscript has been comprehensively revised in response to the recommended modifications.Please refer to the “Response to Reviewers-4“， " Revised Manuscript with Track Changes-4" and " Manuscript-4" for further details.

**Question 2.** If you would like to make changes to your financial disclosure, please include your updated statement in your cover letter.

**Answer:** The financial disclosure statement remains unchanged. The statement of financial disclosure can be seen in “cover letter-4”.

**Question 3.** 1. If the reviewer comments include a recommendation to cite specific previously published works, please review and evaluate these publications to determine whether they are relevant and should be cited. There is no requirement to cite these works unless the editor has indicated otherwise.

**Answer:** The articles recommended by the reviewers were reviewed, confirming their relevance to this study and suitability for citation. See Ref. 2 under "References", as can be seen at lines 50 and 410.

**Question 4.** Please review your reference list to ensure that it is complete and correct. If you have cited papers that have been retracted, please include the rationale for doing so in the manuscript text, or remove these references and replace them with relevant current references. Any changes to the reference list should be mentioned in the rebuttal letter that accompanies your revised manuscript. If you need to cite a retracted article, indicate the article’s retracted status in the References list and also include a citation and full reference for the retraction notice.

**Answer:** References 13 and 22, which were retracted or controversial, have been removed and replaced with relevant existing literature. This can be found in lines 442-446 and 474-479 of the “Revised Manuscript with Track Changes-4”. The revised reference list no longer includes "withdrawn or controversial references". Additionally, reference 35 was removed due to its duplication of reference 15, with subsequent reference numbers renumbered accordingly. This can be found in lines 382-384 and 516-518 of the “Revised Manuscript with Track Changes-4”. And more, minor revisions have been made to the format of all references in accordance with the template requirements. This can be found in lines 409-542 of the “Revised Manuscript with Track Changes-4”.

**Question 5.** If applicable, we recommend that you deposit your laboratory protocols in protocols.io to enhance the reproducibility of your results.

**Answer:** After discussion with the staff of Platform protocols.io, this experimental protocol is deemed unsuitable for implementation on Platform protocols.io. Therefore, this experimental protocol has not been deployed on Platform protocols.io. However, all raw data generated from this experiment have been fully disclosed within the manuscript as “File S2”.

**2** **Questions Raised by Reviewer 3**

**Question 1.**They replaced the text "Chairs of landmarks" with "Waiting chairs" in the "Lobby" part of the figure. However, the text "Chairs of landmarks" in the "Waiting room" still needs to be changed.

**Answer:** The “chairs of landmarks” has changed into “Landmarks” and “Waiting chairs”,as can be seen in the "Waiting room" of figure 4. Furthermore, we have further refined Figure 4 by integrating the pathway for nuclear medicine technologists (depicted by yellow arrows in the diagram) and increase explanatory text in the caption of Figure 4: " Blue arrows depict the patient's route, while yellow arrows denote the nuclear medicine technologist's pathway." This can be found in lines 145-146 and 549-550 of the “Revised Manuscript with Track Changes-4”.

These are my comprehensive responses. I eagerly await your feedback.

Best wishes!

Dandan Shang

2026.1.19
